# When is a GIST not a GIST? A case report of synchronous metastatic gastrointestinal stromal tumor and fibromatosis

**DOI:** 10.1186/1477-7819-7-8

**Published:** 2009-01-21

**Authors:** Chee Khoon Lee, Alison Hadley, Keshani Desilva, Gareth Smith, David Goldstein

**Affiliations:** 1 Department of Medical Oncology, Prince of Wales Hospital, NSW, Australia; 2 Department of Medical Oncology, St George Hospital, NSW, Australia; 3 Department of Anatomical Pathology, Pacific Laboratory Medicine Services, NSW, Australia; 4 Department of Surgery, Royal North Shore Hospital, NSW, Australia

## Abstract

**Background:**

A number of non-malignant diseases that share similar morphological features as gastrointestinal stromal tumor (GIST) have been reported. Co-existence of GIST with these other diseases is rarely recognized or reported.

**Case presentation:**

We report a case of a 62 year-old man with long-term stable control of metastatic GIST with systemic therapy, presented with an apparent intra-abdominal progression but not supported by imaging with positron emission tomography. Subsequent resection of the intra-abdominal tumor identified a non-malignant fibroid.

**Conclusion:**

Differentiating localized progression of GIST from other diseases has important prognostic and therapeutic implications. The potential for co-existence of non-malignant soft tissue neoplasm should always be considered.

## Background

The finding of gain-of-function mutation of KIT has revolutionized the treatment of advanced gastrointestinal stromal tumor (GIST). This has subsequently led to development of effective systemic therapy utilizing tyrosine kinase inhibitors (TKI). Imatinib is the prototype TKI that was initially reported to achieve a partial response rate of 53.7% and stable disease rate of 27.9%[1]. With the increasing use of TKI in the treatment of advanced GIST, the pattern of disease evolutions are changing which will ultimately impact on the approach to management.

A number of soft tissue neoplasm share many similarities in the morphological and immunophenotypic profiles with GIST. Aggressive fibromatosis (AF) and keloid type fibromatosis scar tissues are distinct soft tissue tumors. AF is a fibroproliferative disease with a propensity for intra-abdominal presentation[2]; it may be locally aggressive but generally lacks metastatic potential. Keloid and hypertropic scars are closely related entities that are non-malignant and characterized histologically by increased connective tissue deposition, increased blood vessel density and increased cellular deposition[3,4].

In this report, we present a case of a man who had been treated with imatinib and achieved long-term stable advanced GIST, but presented with localized proliferation of soft-tissue neoplasm mimicking GIST.

## Case presentation

**Figure 1:**
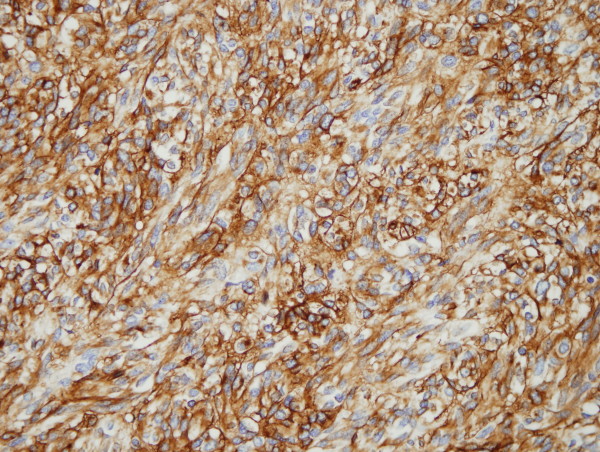
**A representative immunohistochemical section of the resected primary tumor – diffuse c-KIT staining**.

A previously healthy, 62 year old man was diagnosed with a gastric antral tumor after investigations for symptomatic anemia. A barium swallow confirmed the presence of tumor causing subacute gastric outlet obstruction. Laparoscopy identified a gastroduodenal tumor and synchronous bilobar liver metastases. No peritoneal disease was identified. The primary tumor was completed excised and a liver biopsy was performed intra-operatively. Histopathology was consistent with metastatic malignant gastrointestinal stromal tumor, with typical spindle cell features on light microscopy. C-KIT was positive and the mitotic rate was 60/50 per high power fields (Figure 1). Subsequent analysis of the tumor revealed an in-frame deletion of Exon 11 in the C-KIT gene.

Post-operatively, this man recovered well from his surgery and was commenced on imatinib 600 mg daily; he subsequently required dose-reduction to 400 mg daily due to grade 3 neutropenia. He still had residual hepatic metastatic disease which was visible on computerized tomography [CT] scan, and was FDG-avid on positron emission tomography [PET] evaluation. For a period of eighteen months after surgery, imatinib was tolerated well and achieved good disease control. CT and PET imaging during this period revealed regression of size of the liver metastases. After two years of therapy, however, a CT scan revealed an increase in size of a dominant segment VI hepatic metastasis which was treated with radiofrequency ablation. He was then maintained on imatinib at 600 mg daily with subsequent disease control.

**Figure 2:**
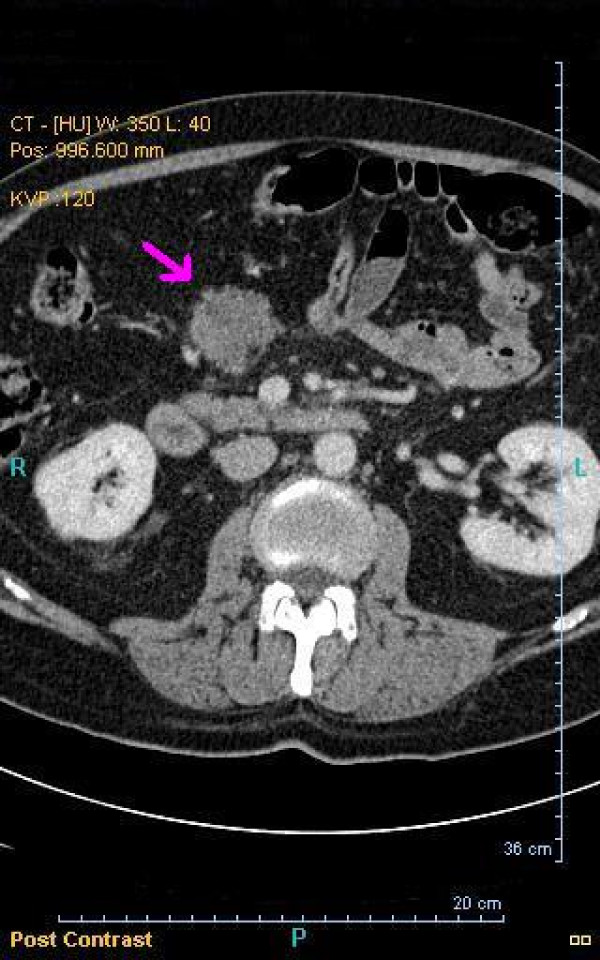
**A section of computerized tomography [CT] scan**. Arrow identifies the infrapyloric mesenteric mass.

**Figure 3:**
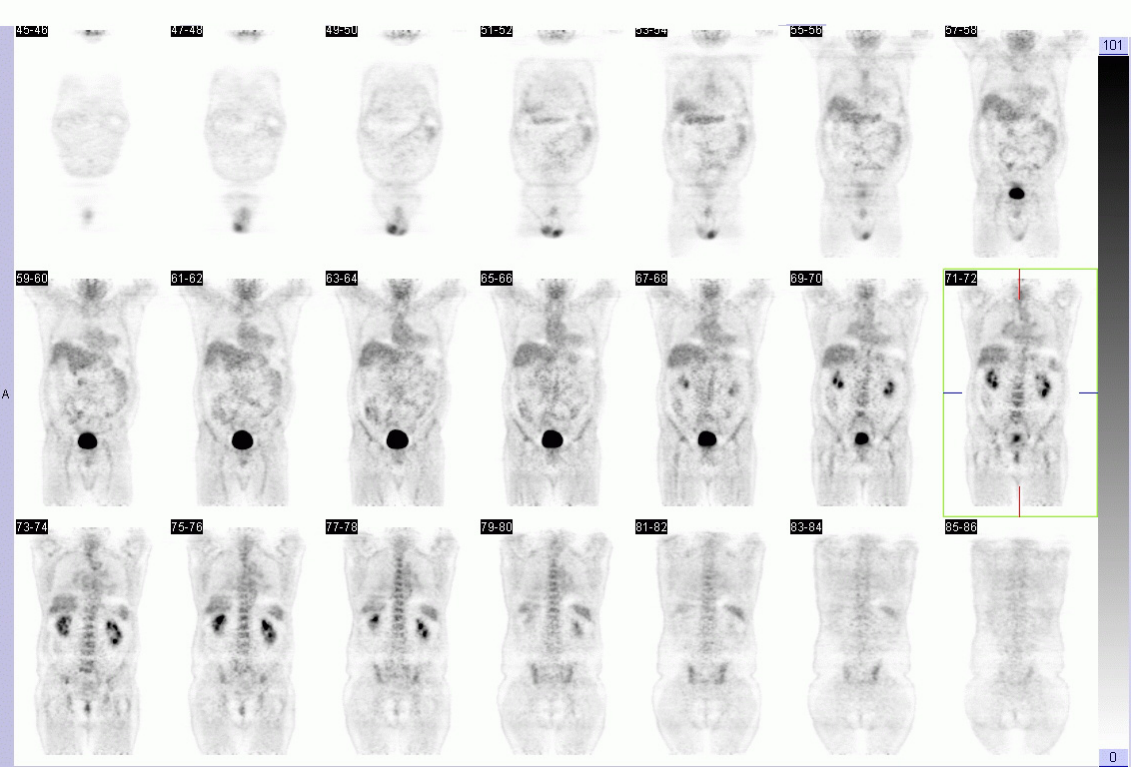
**Whole body positron emission tomography [PET]**. No abnormal foci of increased metabolism of FDG can be identified.

At four and half years from diagnosis, an asymptomatic infrapyloric mesenteric mass was identified on a surveillance CT which progressively increase in size over the next two months (Figure 2). A PET scan paradoxically revealed no glucose avidity of this mesenteric tumor (Figure 3).

**Figure 4:**
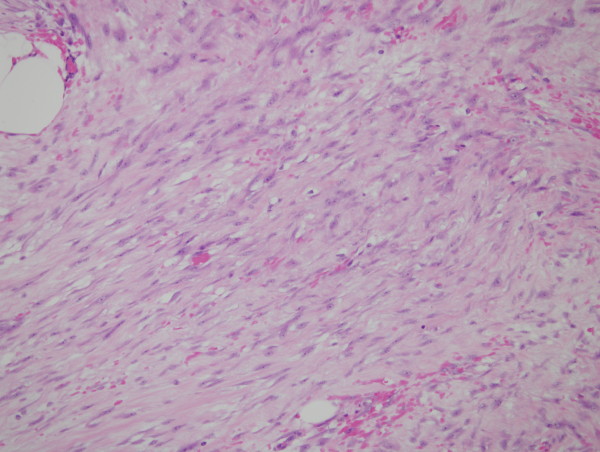
**Hematoxylin & eosin stained section of infrapyrolic mesenteric mass**. Spindle shaped fibrocytic/fibroblastic like cells amongst intervening collagen.

At subsequent laparotomy, the tumor was found to be lying within the peritoneal leaves of the mesocolon extending from the origin of the superior mesenteric vessels to the inferior pancreatico-duodenal vessels. Histopathology showed a tumour mass composed of spindle shaped fibrocytic/fibroblastic like cells amongst intervening collagen (Figure 4) with low mitotic rate (less than 1 per 50 hpf). Immunoperoxidase staining was positive for C-KIT but negative for CD34 and S100. Genetic analyses did not identify the previous C-KIT Exon 11 in-frame deletion or mutations of other Exons 11, 9, 13 and 17 and PDGFRA Exon 18.

Imatinib was continued 600 mg daily, with brief cessation during the peri-operative period, as metastatic GIST remained radiologically stable. No specific adjuvant therapy for the soft-tissue tumor was employed post-operatively.

## Discussion

In this patient with metastatic GIST, the development of the mesenteric tumor four years after the institution of imatinib initially suggested disease relapse. Debulking surgery remains a recognized standard practice in the case of local progression where such procedure is associated with prolonged survival with the elimination of imatinib resistance clones[5]. However, in rare instances as illustrated in this case, consideration for co-existence of another disease will need to be considered.

This patient underwent surgery with the pre-surgical diagnosis of a localized progression; surgery was aimed to achieve disease control with the elimination of a presumably localized imatinib resistance tumor. Post-surgery, the histopathologic findings revealed a tumor with reduced cellularity and low mitotic activity consistent with the pre-operative non-glucose avid PET findings. Collaborative pathologic review was obtained and excluded diagnosis of a recurrent GIST. However, a definitive uniform diagnosis could not be made. The possible differential diagnoses of this soft-tissue tumor include aggressive fibromatosis (AF) or intra-abdominal keloid type fibrocollagenous scar.

Immunohistochemistry was positive for C-KIT, which is unusual in AF or intra-abdominal keloid type fibrocollagenous scar. Mutation analysis that was performed on the mesenteric tumor further clarify that this mass, which was absent of Exon 11 C-KIT mutation, was different from the Exon 11 C-KIT mutation positive of the original resected antral GIST.

Histologically, AF lies on a spectrum of disorders characterized by excess proliferation of fibroblast-like spindle cells[6]. These cells are monoclonal neoplasms[7] with low cellularity and rare mitoses. Most are associated with germline or somatic mutations of WNT pathway (*APC *or *CTNNB1*). Some studies [8-11] have demonstrated clinical and radiological benefits of imatinib in treatment of AF.

There is very limited literature on intra-abdominal keloid type fibrocollagenous scar. The scar in this patient was presumably formed from previous surgical laparotomy. There is growing evidence to suggest that Transforming Growth Factor β[12] is implicated in keloids and other benign fibroproliferative diseases as well as formation of adhesions after abdominal operations[13]. Although such clinical entities are well-described in literature when they are manifested cutaneously, there is no information on intra-abdominal manifestation.

The immunophenotypic profiles of GIST, AF and keloid may overlap. Fibromatoses may stain for vimentin, smooth muscle actin, and desmin. In some series, fibromatosis did not stain for CD34 or S-100 protein, while CD34 staining and occasional S-100 protein positivity were seen in GIST[2,14]. It has been suggested that differences in CD34 immunostainin might be helpful in distinguishing between them[14]. C-KIT staining in AF is controversial. Up to 75% of cases in one series were C-KIT positive[2] but other reports have concluded that most AF's do not express demonstrable levels of this imatinib target. In cutaneous fibrocollagenous scar, smooth muscle actin is stained more commonly in hypertropic scar and to the lesser extent on keloid scars[12,15]. More importantly, there is no report of C-KIT staining in fibrocollagenous scars.

## Conclusion

The long stable disease control, the absence of glucose avidity of pre-operative PET and the absence of the C-KIT mutation in the mesenteric tumor were all features that might have suggested the possibility of an alternative diagnosis to GIST recurrence. Although surgical resection of this mesenteric mass may remain necessary, a correct diagnosis has important implication for his future systemic therapy. This case report highlights the importance of recognizing the coexistence of other diseases in patients with chronic GIST.

## Consent

Written informed consent was obtained from the patient for publication of this case report and accompanying images. A copy of the written consent is available for review by the Editor-in-Chief of this journal.

## Competing interests

The authors declare that they have no competing interests.

## Authors' contributions

CL and AH drafted the original manuscript, with subsequent further contributions from KD, GS and DG. KD reported the histopathological findings and supplied the photomicrographs used in this manuscript. GS reported the surgical findings

All authors read and approved the final manuscript.
